# Impact of Injection Protocol Selection by Retina Specialists on Clinical Outcomes in Patients with Diabetic Macular Edema

**DOI:** 10.3390/life12010051

**Published:** 2021-12-31

**Authors:** Anika Tanwani, Nida Safdar, Amir Ali, Cina Karimaghaei, Mary Schmitz-Brown, Ahmad Rehmani, Praveena K. Gupta

**Affiliations:** 1 School of Medicine, University of Texas Medical Branch, Galveston, TX 77550, USA; antanwan@utmb.edu (A.T.); nisafdar@utmb.edu (N.S.); amiali@utmb.edu (A.A.); cikarima@utmb.edu (C.K.); 2 Department of Ophthalmology and Visual Sciences, University of Texas Medical Branch, Galveston, TX 77550, USA; meschmit@utmb.edu (M.S.-B.); ahrehman@utmb.edu (A.R.)

**Keywords:** diabetic macular edema, intravitreal injections, best-corrected visual acuity, central macular thickness, anti-VEGF, injection protocol

## Abstract

Intravitreal anti-VEGF injections are the current gold standard for treating diabetic macular edema (DME). However, injection practice patterns of retina specialists have varied markedly based on physician discretion. This retrospective study analyzes the impact of injection protocol selection on change in best-corrected visual acuity (BCVA) and central macular thickness (CMT) in 170 eyes treated by 4 retina specialists practicing a pro re nata (PRN) strategy between 2010 and 2020. DME patients received an average of 7.25 injections every 6.24 weeks over 56.6 weeks. There were significant differences between retina specialists in mean number of injections (*p* = 0.0001) and mean length of treatment (*p* = 0.0007) but not in mean interval between injections. Over the treatment period, average change in BCVA was −0.053 logMAR, and average change in CMT was −51.1 µm, neither of which had significant differences between retina specialists. BCVA and CMT at initial visit were found to be significantly associated with improved BCVA and CMT over the treatment period (*p* < 0.001). Number of injections administered and interval between injections were not found to be significant factors affecting change in BCVA or CMT. Despite significant differences in injection dosing regimen, retina specialists achieved similar outcomes in change in BCVA and CMT over the treatment period.

## 1. Introduction

Diabetes mellitus (DM) currently affects 463 million adults worldwide, with an expected rise to 700 million by the year 2045 [1]. DM may have many ocular manifestations including diabetic retinopathy and diabetic macular edema (DME). DME is the leading cause of vision loss in working age adults with DM [2]. Chronic hyperglycemia instigates microangiopathy and degenerative neuroretinopathy through weakening of the blood-retinal barrier from pericyte loss [3]. The resulting endothelial dysfunction leads to release of vascular endothelial growth factor (VEGF), which in turn incites capillary leakage resulting in a collection of extracellular fluid in the macula, or DME [4,5,6,7]. Historically, diabetic macular edema has been treated with laser photocoagulation [8]. However, advancements in pharmacological therapies, particularly intravitreal VEGF inhibitors, have exhibited significant improvements in macular edema anatomically as well as visual recovery [9]. Hence, anti-VEGF therapy has become the gold standard treatment for most patients with DME. The three specific anti-VEGF drugs commonly used for treatment of DME include bevacizumab, ranibizumab, and aflibercept. Ranibizumab and aflibercept are both FDA approved while bevacizumab is used off-label for treatment of DME. Despite these differences, the three anti-VEGF therapies have demonstrated similar efficacy on reduction of retinal thickness and improvement of visual acuity in DME patients with mild vision loss [10].

Because patients with DME represent a heterogeneous group that have varied responses to anti-VEGF therapy, there are no established guidelines on standardized injection protocols for retina specialists to follow. The common clinical injection protocols practiced are fixed monthly injections for the first three months followed by either continuation pro re nata (PRN), in which patients are treated as-needed, or with a treat-and-extend (T&E) strategy, in which treatment interval is incrementally increased until maximal length of quiescence of disease is achieved [11]. In recent years, T&E has been heavily researched in hopes to implement a strict plan for the longest interval without compromising progression of the disease and thereby decreasing the number the number of injections [12,13]. A head-to-head study showed that patients under a PRN regimen received significantly fewer injections than those under a T&E regimen, suggesting PRN strategies may be better for reducing patient burden [14]. However, another study indicated that T&E strategies lead to high patient adherence and visual acuity gains [15]. Both studies reveal that there is a need to standardize intravitreal injection protocols among retina specialists to reduce patient and physician burden.

Frequent intravitreal injections not only carry ophthalmological risks but may also negatively affect the quality of life of the patient. Patients have to worry about practical inconveniences, such as cost of injections, absences from work, and reliance on family members and caregivers to accompany them to the frequent visits, as well as emotional stressors of frustration, treatment anxiety, and needle phobia [16]. Increasing number of injections also poses a risk for adverse physical effects such as endophthalmitis, intraocular inflammation, intraocular pressure elevation, or ocular hemorrhage [17]. DM itself is already associated with additional comorbidities such as hypertension, hypercholesterolemia, chronic kidney disease, and ischemic heart disease. These may lead to additional burdensome complications such as undergoing limb amputation or regular dialysis treatment that further exacerbate the emotional stress and treatment fatigue felt by DME patients [18].

Injection protocols of retina specialists have varied markedly based on physician discretion, patient preference, and treatment outcomes. Because of this, there are no established clinical practice guidelines in place for treatment of DME patients. Such heterogeneity in the DME patient population along with large variance among treatment regimens between physicians has led to ambiguity in how to best treat patients. Although multiple studies have analyzed the effects of differences in anti-VEGF drugs on clinical outcomes, the impact of specific injection protocol selection of individual retina specialists has not been studied [9,10,19,20,21,22,23]. This retrospective chart review investigated the efficacy of individualized injection protocols of four different retina specialists utilizing anti-VEGF PRN injection therapy with respect to visual outcome and anatomical changes in the macula. Specifically, the two main outcomes assessed were improvement in best corrected visual acuity (BCVA) and decrease in central macular thickness (CMT) which are clinical markers used to assess progression of DME.

## 2. Materials and Methods

### 2.1. Ethics

Ethical approval was obtained the Institutional Review Board of University of Texas Medical Branch in accordance with the tenets of the Declaration of Helsinki.

### 2.2. Study Design

A retrospective chart review using Epic Systems Electronic Medical Records were used to identify patients with ICD codes E11.321, E11.331, E11.341, and E11.351 for clinically diagnosed DME with visits between January 2010 and July 2020 by retina specialists practicing at University of Texas Medical Branch, Galveston. All practicing retina specialists were trained in Retina Fellowships with Accreditation Council for Graduate Medical Education (ACGME) accreditation in the United States.

Patient inclusion criteria included diagnosis of DME, age 18–100 years, and administration of aflibercept, ranibizumab, and bevacizumab in a PRN pattern. Exclusion criteria included patients with coexisting conditions that complicated the evaluation of DME, including wet AMD, retinal artery occlusion, retinal vein occlusion, high myopia (>6 diopters), trauma, and history of anti-VEGF injections or laser photocoagulation. Patients with no CMT data available during the treatment period, patients who switched between retina specialists, and patients whose physicians treated fewer than five patients over the treatment period were also excluded. Each qualified eye was counted separately for analysis.

All patients initially received loading doses of monthly anti-VEGF injections for three consecutive months after which retina specialists proceeded with an individualized PRN regimen. Patient demographics, comorbid conditions, total number of injections, time interval between injections, and BCVA and CMT at each injection visit were collected. The mean CMT obtained from Heidelberg Spectralis ocular coherence tomography (OCT) for each eye was retrieved up to 30 days before and after starting treatment for each eye. CMT is defined as the mean thickness measured at the point of intersection of the 6 radial scans on optical coherence tomography. Comorbid conditions including smoking history, chronic kidney disease, hyperlipidemia, hypertensive retinopathy, and pseudophakia were also collected for each patient. BCVA was recorded as lines from Snellen chart and converted to logMAR. Changes in BCVA and CMT over the treatment period were calculated as the difference between these values from the first and last injection (last injection–first injection).

### 2.3. Statistical Analyses

Statistical analyses were performed to determine the relationship between physician injection protocols and changes in BCVA and CMT over the treatment period using Chi-square tests for categorical data and Kruskal–Wallis tests for continuous data. Multivariate generalized linear modeling was utilized to determine factors associated with achieved difference in visual acuity and macular thickness over course of injection treatment.

## 3. Results

Epic Systems EMR identified 176 clinically diagnosed DME patients that met the inclusion criteria seen between January 2010 and July 2020 by 8 identified retina specialists. Out of 176 patients, 52 patients were excluded due to being treated by multiple retina specialists. Four retina specialists were excluded because they had fewer than five DME patients over the time frame, which subsequently resulted in the exclusion of 2 additional patients from the study. A total of 170 eyes from 122 DME patients between 4 retina specialists labeled as A, B, C, and D were included in the data analysis.

### 3.1. Demographics

The mean age of the 122 included DME patients was 62.2 ± 11.3 years, who were equally divided in gender with 61 males (50%) and 61 females (50%). As far as racial distribution, 83 patients (68.03%) were Caucasian, 38 (31.15%) were African American, and 1 (0.82%) was Asian. Assessment of comorbidities and medical history revealed 35 (28.69%) were smokers, 62 (50.82%) had chronic kidney disease, 92 (75.41%) had hyperlipidemia, and 38 (31.15%) had hypertensive retinopathy (Table 1).

### 3.2. Injection Protocol Selection in PRN Treatment Regimens among Retina Specialists

Looking at anti-VEGF drug preferences, 1 drug was used in 83.3, 90.9, 78.4 and 96.8% of patients for retina specialists A, B, C, and D respectively (*p* = 0.07). The remaining patients received a combination of 2 or 3 drugs in no defined pattern. With respect to choice of specific anti-VEGF drug, for retina specialists A, B, C, and D, respectively, bevacizumab was used most frequently in 100, 95.5, 100 and 100% of patients (*p* = 0.08), followed by aflibercept for 16.7, 4.6, 20.7 and 3.2% of patients (*p* = 0.046), and ranibizumab least frequently for 0, 9.1, 2.7 and 0% of patients (*p* = 0.26) (Table 2).

**Figure 1:**
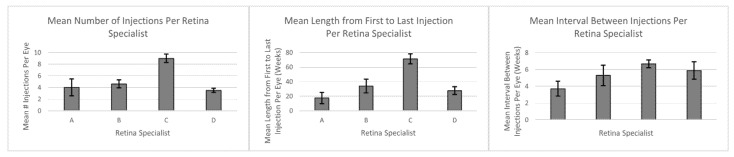
Graphical representation of intravitreal injection protocols between retina specialists.

As far as distribution of treated eyes, retina specialist A treated 6 eyes, retina specialist B treated 22 eyes, retina specialist C treated 111 eyes, and retina specialist D treated 31 eyes. The mean number of injections given per eye was 4.0 ± 1.44, 4.6 ± 0.69, 9.0 ± 0.71, and 3.5 ± 0.34 for retina specialists A, B, C, and D, respectively, with an overall average of 7.25 injections (*p* = 0.036). The mean length of injection treatment in weeks was 17.57 ± 7.67, 34.00 ± 9.33, 71.29 ± 6.94, and 27.57 ± 5.31 for retina specialists A, B, C, and D, respectively, with an overall average of 56.6 weeks (*p* = 0.0007). The mean interval period between injections in weeks was 3.7 ± 0.89, 5.29 ± 1.21, 6.67 ± 0.47, and 5.87 ± 1.03 for retina specialists A, B, C, and D, respectively with an overall average of 6.24 weeks; however, these differences were not statistically significant (*p* = 0.381) (Table 3, Figure 1).

### 3.3. Primary Outcome—Change in Best Corrected Visual Acuity (BCVA)

**Figure 2:**
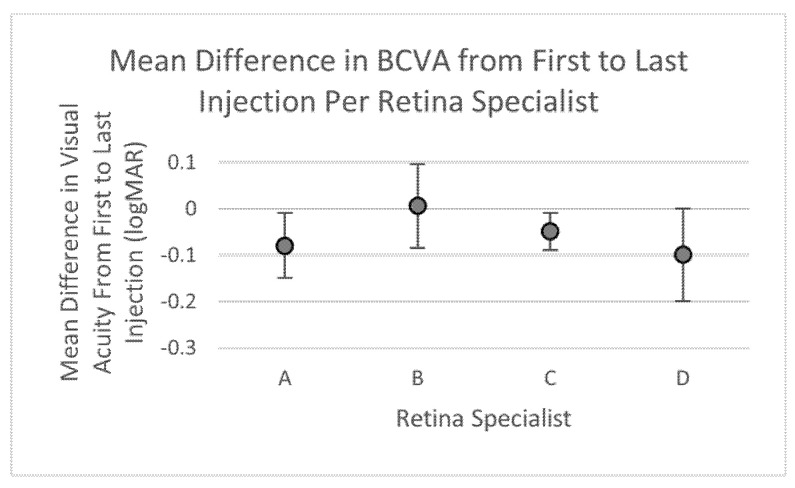
Graphical representation of mean change in BCVA over treatment period per retina specialist.

The change in BCVA from first to last injection visit was the primary outcome used to assess treatment efficacy. The mean BCVA at first treatment in logMAR units was 0.66 ± 0.2, 0.73 ± 0.12, 0.59 ± 0.05, and 0.92 ± 0.12 for retina specialists A, B, C, and D, respectively, with an overall average of 0.67 logMAR, which significantly differed between retina specialists (*p* = 0.036). However, the mean difference in BCVA from first to last injection measured in logMAR units was −0.08 ± 0.07, 0.005 ± 0.09, −0.05 ± 0.04, and −0.1 ± 0.11 for retina specialists A, B, C, and D, respectively, with an overall average of −0.053 logMAR, and differences were found not to be significant (*p* = 0.226) (Table 4, Figure 2).

Multivariate generalized linear modeling was used to assess which factors played a significant role in change in BCVA from first to last visit. Factors included in analysis were BCVA at first visit, retina specialist, age, sex, race, smoking status, number of injections, interval between injections, hyperlipidemia, and hypertensive retinopathy. Retina specialist B was used as the reference to highlight differences compared to other retina specialists which will be discussed in the next section. The analysis showed that the only factor that played a significant role in change in BCVA was BCVA at first visit, which had an estimated effect size of −0.32 (*p* < 0.001) (Table 5).

### 3.4. Secondary Outcome—Change in Central Macular Thickness (CMT)

**Figure 3:**
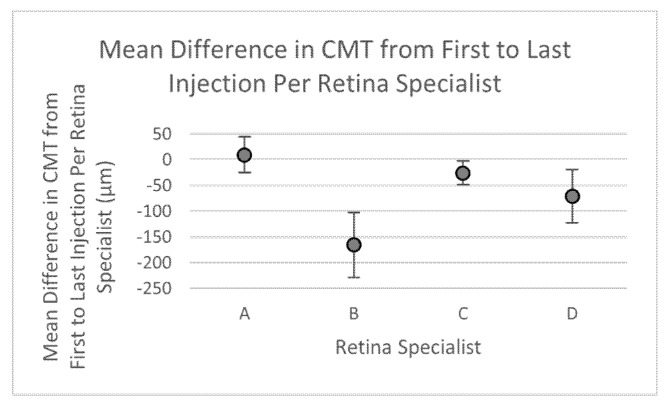
Graphical representation of mean change in CMT over treatment period per retina specialist.

The secondary outcome used to assess treatment efficacy was change in CMT from first to last injection visit. The mean CMT at first visit was 342.5 ± 62 μm, 416 ± 63.1 μm, 327 ± 18.2 μm, and 429 ± 50.4 μm for retina specialists A, B, C, and D, respectively, with an overall average of 357.5 μm and was not significantly different among retina specialists (*p* = 0.131). Mean difference in CMT from first to last injection visit was 9.3 ± 35.1 μm, −165.8 ± 63.2 μm, −26.0 ± 22.5 μm, and −71.4 ± 51.9 µm for retina specialists A, B, C, and D, respectively, with an overall average of 51.1 µm and was also not significantly different amongst the four retina specialists (*p* = 0.06) (Table 6, Figure 3).

Multivariate generalized linear modeling was additionally used to determine which factors played a significant role in change in CMT from first to last visit. Factors included in analysis were CMT at first visit, retina specialist, age, sex, race, smoking status, injection number, interval between injections, hyperlipidemia, and hypertensive retinopathy. Retina specialist B was made to be the reference to highlight significant differences compared to the other retina specialists. The analysis showed that compared to retina specialist B, retina specialists A, C, and D showed significant differences in terms of their effect on change in CMT with effect estimates of 151.34, 81.65, and 114.20 and *p* of 0.03, 0.03, and 0.01, respectively. Caucasian race is also a factor shown to be significant in this model with an effect estimate of 58.38 (*p* = 0.05) when compared to other races as the reference (Table 7).

## 4. Discussion

We provide a comparison of anti-VEGF PRN treatment regimens between four retina specialists with respect to change in BCVA and CMT in DME patients. While there are multiple published studies comparing the efficacy of the different anti-VEGF drugs on the treatment of DME, a “head-to-head” comparison of injection protocols between retina specialists has never been done [9,10,19,20,21,22,23]. The lack of “generalized practice guidelines” in intravitreal injection protocol among retina specialists could be due to the heterogenous multifactorial nature of the biology of DME manifestations and industry standard controls of different anti-VEGF drugs.

This study first categorically compared treatment regimens with respect to the choice of anti- VEGF drug, the number of anti-VEGF injections, and the intervals between injections used by retina specialists. Of note, there was a significant difference in aflibercept use between the retina specialists; however, bevacizumab and ranibizumab were administered similarly (Table 2). This differential utilization of aflibercept use may be because this drug was not approved for the treatment of DME until July 2014 instead of practitioner preference for this drug in DME treatment [24]. Conventionally, bevacizumab is used as initial therapy and is subsequently switched to ranibizumab or aflibercept for patients with refractory disease, which could also explain a differential choice in use of anti-VEGFs by the retina specialists [25]. These differences in use of aflibercept may also lead to differences in improvements in BCVA as some patients have been shown to have larger gains in vision with aflibercept than with bevacizumab or ranibizumab treatment, particularly patients with worse starting BCVA [10]. Evidence also suggests that ranibizumab has shown the same or better efficacy than bevacizumab in the treatment of DME [25,26]. Significant differences were also found when comparing mean number of intravitreal injections per eye and the mean length from first to last injection between the retina specialists. However, the mean interval between intravitreal injections was found to be statistically similar between retina specialists (Table 3, Figure 1). This shows that there are differences in some facets of the treatment regimens between the retina specialists.

We further explored whether the injection protocols used by the retina specialists impacted changes in BCVA and CMT in clinical outcome of DME management. At baseline before anti-VEGF treatment was initiated, visual acuities of the DME patients were significantly different among the four physicians. However, baseline CMTs of patients were statistically similar amongst the four retina specialists suggesting an anatomical consensus in when to initiate treatment. Despite differences in protocols of treatment regimens and initial BCVA, both mean changes in BCVA and CMT from first to last injection were statistically similar amongst the retina specialists (Table 4 and Table 6, Figure 2 and Figure 3). Such findings are corroborated by ECHO study report 1 which found that an increased number of injections did not necessarily lead to improvements in BCVA, and although central retinal thickness (CRT), defined as retinal thickness in the central subfield, appeared to improve somewhat at first, continual improvement in mean CRT with more injections was not seen [27]. After conversion to ETDRS (Early Treatment Diabetic Retinopathy Study) scale, the combined average change in BCVA amongst all four retina specialists was +2.653 letters. This value was lower than previously published data in trials such as RISE, RIDE, DRCR.net Protocol I, RESTORE, RESOLVE, and Protocol T that demonstrated BCVA gains ranging from +6.1 to +13.3 letters in one year [10,20,22,23,28,29]. Additionally, in the RISE and RIDE trials, mean CRT continually improved through 12 months of monthly ranibizumab injections [22]. This contrasts with results from our study, which has an average CMT improvement of 51.1 μm with no significant differences in CMT changes between providers despite differences in number of injections. Differences might be explained by the strict monitoring of adherence that is required by clinical trials which leads to increased patient compliance when compared to the real-world. As a result, these trials may fail to represent actual clinical practice well enough to be generalizable to large populations [30]. Comparative discrepancies in results may occur as a result of study design, inclusion-exclusion criteria, anti-VEGF choices, and provider-patient compliance.

Based on the multivariate analysis, BCVA at first visit was the only variable that played a significant role in affecting change in BCVA over the treatment period. CMT at first visit, retina specialist, and race demonstrated significance in determining change in CMT over the treatment period. In agreement with previously published work, this study demonstrates that worse vision and thicker central macula at baseline is associated with more improvement in BCVA and CMT over time [31,32]. This is likely due to the ceiling effect, which occurs when an independent variable no longer affects the dependent variable [19]. Of note, the number of injections and intervals between injections were not found to be significant in determining the final change in BCVA or CMT. In evaluating race, Caucasian patients were shown to have significantly worse outcomes in CMT improvement, which contrasts with previous analyses that have revealed that there were no statistically significant differences in reduction of CMT between race groups [33]. This finding may be due to certain subsections of Caucasian patients suffering from more advanced disease causing further ophthalmologic impairment. However, future analysis will need to be conducted to ascertain whether this hypothesis holds true.

Although patients treated by retina specialist B demonstrated the greatest improvement in CMT, they saw no improvement in BCVA on average. Other investigations have found that reduction of CMT has an inconsistent and weak association with outcome of BCVA; specifically, studies have shown a lag in potential visual improvement behind resolution of macular edema [28,29,34]. Inversely, other studies have also shown that visual gain does not consistently equate with anatomical recovery in the macula due to secondary causes, such as structural defects of photoreceptors, chronic ischemia-induced malfunction of the blood–brain barrier, neural apoptosis, and glial reactivity which may worsen anatomy but not vision [35]. This aligns with what was seen with retina specialist A who exhibited improvement in BCVA but not in CMT.

Administration of intravitreal injections poses significant clinical, humanistic, and economic burden on patients. Each recommended monthly visit requires a comprehensive ophthalmic exam, fundoscopy, OCT imaging, and intravitreal injections [36]. Patients reported spending an average of 12.5 h per month, or 150 h annually, on appointments [37]. Diabetic patients already require on average more than 25 outpatient visits a year with a large majority of these being non-ophthalmologic, so additional monthly appointments for intravitreal injections further increase this burden [38]. These visits are also anxiety-provoking as understandably many patients are fearful of needle penetration into the eye, further exacerbated by travel time and long waits in clinics [39]. Financially, aflibercept and ranibizumab range from 1800–2000 USD per injection, and bevacizumab is about 50 USD per injection [40]. This does not take into account costs of appointments and diagnostic tests. Many patients already experience loss of income due to the visual handicaps that are accompanied by DME, so high treatment costs can be detrimental. Reduction in the frequency of injections could not only diminish the burden of therapy but also increase compliance rates. A survey study focusing on DME patients found that 95% of respondents felt that lessening frequency of injections and appointments in general for equivalent visual results would be the most efficacious change for reducing their treatment burden [37]. Recent studies have shown that patient compliance with anti-VEGF treatment is likely not as regular or frequent as suggested by standards established by clinical trial data [41,42,43,44]. Since DME patients already have a significant healthcare burden due to their diabetes, considering intensity of treatment is critical when creating a therapeutic plan. Thus, this study highlights that interval of injections can be standardized with the intention of keeping longer intervals between injections without affecting patient outcomes. These changes would lower injection burden to patients, caregivers, and healthcare institutions, an important consideration as the prevalence of DM continues to rise.

Like any other retrospective study, there are some limitations in study design. Small sample size, unequal subject distribution, retrospective nature, and missing data points may have influenced our observation in treatment outcomes. Furthermore, the sample size of retina specialists was also small with unequal patient distribution amongst retina specialists, likely leading to variances in data. Confounding variables that may have affected outcomes such as use of intravitreal steroids or poor systemic control and duration of diabetes were not considered and should be included in future studies. However, the strength of our study is the availability of four retina specialists in a single hospital-based setting where intravitreal injection clinics are controlled by the university’s strict guidelines. Therefore, every retina specialist abided by the same compliance policy with minimum influence from payors.

In summary, this study revealed that despite significant differences in number of anti-VEGF injections administered and overall length of treatment of DME patients, all four retina specialists had similar outcomes with respect to changes in BCVA and CMT. Additionally, both baseline BCVA and CMT were found to be significant in determining change in BCVA and CMT, respectively, while number of injections and interval between injections were not. An extended large scale prospective study with incorporation of other co-factors that could affect clinical decision making of intravitreal injections may help to explore further possibility of having a standardized intravitreal injection regimen for DME treatment.

## Figures and Tables

**Table 1:** Baseline patient demographics and medical history.

Characteristics	Category	Value (%)
Age		62.2 (11.3)
Sex	Female	61 (50)
	Male	61 (50)
Race	American Indian/Alaskan Native	0 (0)
	Asian	1 (0.82)
	Black/African American	38 (31.15)
	Caucasian/White	83 (68.03)
Smoking		35 (28.69)
CKD		62 (50.82)
Hyperlipidemia		92 (75.41)
Hypertensive retinopathy		38 (31.15)

Data are presented as mean (SD) for continuous data and N (%) for categorical data.

**Table 2:** Choice of anti-VEGF drug among retina specialists.

	Specialist A N = 6	Specialist B N = 22	Specialist C N = 111	Specialist D N = 31	*p*-Value
1 drug	5 (83.3)	20 (90.9)	87 (78.4)	30 (96.8)	0.07
2 or 3 drugs	1 (16.7)	2 (9.1)	24 (21.6)	1 (3.2)	
Bevacizumab	6 (100)	21 (95.5)	111 (100)	31 (100)	0.08
Aflibercept	1 (16.7)	1 (4.6)	23 (20.7)	1 (3.2)	0.046 *
Ranibizumab	0	2 (9.1)	3 (2.7)	0	0.26

Data are presented as N (%); * for *p* < 0.05.

**Table 3:** Intravitreal injection practice patterns among retina specialists.

	Specialist A N = 6	Specialist B N = 22	Specialist C N = 111	Specialist D N = 31	Overall Average	*p*-Value
Mean number of injections per eye	4 (1.44)	4.6 (0.69)	9.0 (0.71)	3.5 (0.34)	7.25	0.0001 ***
Mean length from first to last injection per eye (in weeks)	17.57 (7.67)	34 (9.33)	71.29 (6.94)	27.57 (5.31)	56.60	0.0007 ***
Mean interval between injections per eye (in weeks)	3.7 (0.89)	5.29 (1.21)	6.67 (0.47)	5.87 (1.03)	6.24	0.381

Data are presented as mean (SD), *** for *p* < 0.001.

**Table 4:** Visual acuity outcomes quantified by BCVA among retina specialists.

	Specialist A N = 6	Specialist B N = 22	Specialist C N = 111	Specialist D N = 31	Overall Average	*p*-Value
Mean BCVA at first treatment (logMAR)	0.66 (0.2)	0.73 (0.12)	0.59 (0.05)	0.92 (0.12)	0.67	0.036 *
Mean change in BCVA over treatment period (logMAR)	−0.08 (0.07)	0.005 (0.09)	−0.05 (0.04)	−0.1 (0.1)	−0.053	0.226

Data are presented as mean (SD), * for *p* < 0.05.

**Table 5:** Multivariate linear regression analysis showing role of different factors on change in BCVA over the treatment period.

Variable	Category	Estimate	*p*-Value
BCVA at first visit		−0.32	<0.001 ***
Retina specialist	A	−0.08	0.68
	C	−0.08	0.47
	D	−0.07	0.58
	B	ref	ref
Age		0.00	0.47
Sex	Female	−0.01	0.87
	Male	ref	ref
Race	Caucasian	−0.01	0.89
	Other	ref	ref
Smoking	Yes	−0.01	0.92
	No	ref	ref
Injection number		−0.01	0.18
Interval between injections		0.00	0.17
Hyperlipidemia	Yes	−0.06	0.43
	No	ref	ref
Hypertensive retinopathy	Yes	−0.01	0.85
	No	ref	ref

*** for *p* < 0.001.

**Table 6:** Anatomical outcomes quantified by CMT among retina specialists.

	Specialist A N = 6	Specialist B N = 22	Specialist C N =111	Specialist D N = 31	Overall Average	*p*-Value
Mean CMT at first measurement (µm)	342.5 (62)	416 (63.1)	327 (18.2)	429 (50.4)	357.7	0.131
Mean change in CMT over treatment period (µm)	9.3 (35.1)	−165.8 (63.2)	−26.0 (22.5)	−71.4 (51.9)	−51.1	0.06

Data are presented as mean (SD).

**Table 7:** Multivariate linear regression analysis showing role of different factors on change in CMT over the treatment period.

Variable	Category	Estimate	*p*-Value
CMT at first visit		−0.88737	<0.001 ***
Retina specialist	A	151.34	0.03 *
	C	81.65	0.03 *
	D	114.20	0.01 **
	B	ref	ref
Age		0.06	0.96
Sex	Female	22.06	0.43
	Male	ref	ref
Race	Caucasian	58.38	0.05 *
	Other	ref	ref
Smoking	Yes	−19.21	0.51
	No	ref	ref
Injection number		0.26	0.92
Interval between injections		−0.46	0.19
Hyperlipidemia	Yes	−16.39	0.57
	No	ref	ref
Hypertensive retinopathy	Yes	−20.41	0.46
	No	ref	ref

* for *p* < 0.05, ** for *p* < 0.01, *** for *p* < 0.001.

## Data Availability

Data presented in this study are available upon request from the corresponding author.
